# Supplementary material for: A Likelihood Approach for Real-Time Calibration of Stochastic Compartmental Epidemic Models
Source: PLoS Comput Biol. 2017 Jan 17;13(1):e1005257. doi: 10.1371/journal.pcbi.1005257 (PMC5240920; doi:10.1371/journal.pcbi.1005257)
Supplement: S1 File — (TAR.GZ) [file pcbi.1005257.s014.tar.gz › HSPH_Online-SI-Revision/PF08/n10-severe/n10-severe_PF_table8.pdf]

1	R0	{1.68952, 11.8402}	{1.92093, 19.9504}	{{1.77913, 19.7514}, {2.1199, 20.}}	{13.6969, 68.4971}	inf	198.	602.331	{537.113, 671.244}	204.208	0.481488	newly	99.	192.59	{162., 225.}	94.5354	0.285509	Reff	0.571396	0.529201	{0.476162, 0.583915}	8.63119	ar	6872.	9280.43	{8922., 9618.}	35.047
2	R0	{1.71739, 9.62661}	{2.16591, 19.8974}	{{1.99856, 20.}, {2.26806, 20.}}	{26.3219, 106.692}	inf	184.	732.433	{669.961, 802.624}	298.062	0.598369	newly	116.	234.5	{206., 264.}	102.155	0.303685	Reff	0.569658	0.497308	{0.461254, 0.542744}	13.4906	ar	6874.	10 102.2	{9851., 10 322.}	46.962
3	R0	{1.48936, 6.23975}	{1.81146, 13.2484}	{{1.77286, 12.6997}, {1.84776, 13.8505}}	{21.6268, 112.323}	inf	38.	420.98	{380.078, 496.352}	1007.84	1.0419	newly	40.	202.55	{180., 228.}	406.375	0.702488	Reff	0.649957	0.670816	{0.652362, 0.690479}	3.28515	ar	5666.	9561.21	{9393., 9729.}	68.7471
4	R0	{1.62394, 7.51135}	{1.74453, 19.8632}	{{1.70774, 19.5478}, {1.76895, 20.}}	{7.54814, 164.442}	inf	86.	614.747	{544.37, 654.061}	614.822	0.852998	newly	66.	199.72	{177., 218.}	202.606	0.479447	Reff	0.601507	0.583178	{0.567188, 0.599179}	3.80927	ar	6417.	8595.92	{8465., 8729.}	33.9554
5	R0	{1.53632, 8.57652}	{1.7711, 19.9137}	{{1.68659, 19.5461}, {1.86489, 20.}}	{15.2819, 132.188}	inf	148.	633.615	{570.764, 691.347}	328.118	0.630024	newly	112.	206.05	{181., 238.}	83.9732	0.262515	Reff	0.610687	0.57474	{0.492532, 0.622348}	7.12064	ar	6260.	8904.09	{8569., 9306.}	42.2379
6	R0	{1.47166, 5.7434}	{1.82245, 19.9433}	{{1.71369, 19.8043}, {1.92507, 20.}}	{23.8366, 247.239}	inf	52.	648.112	{571.288, 720.972}	1146.37	1.09419	newly	51.	209.77	{176., 239.}	311.314	0.611671	Reff	0.657979	0.597422	{0.543188, 0.638385}	9.42844	ar	5650.	8709.37	{8436., 9090.}	54.1481
7	R0	{1.48179, 8.87369}	{1.58813, 15.8167}	{{1.49608, 14.2326}, {1.71974, 20.}}	{7.21121, 78.2429}	inf	96.	337.095	{253.238, 479.835}	251.14	0.530121	newly	76.	135.57	{111., 167.}	78.3816	0.245121	Reff	0.721187	0.668729	{0.632317, 0.707882}	7.35554	ar	5233.	7818.15	{7354., 8443.}	49.4009
8	R0	{1.53669, 6.47807}	{1.90617, 16.5184}	{{1.77631, 13.2318}, {2.00971, 18.2961}}	{24.0441, 154.989}	inf	51.	523.006	{359.66, 591.716}	925.503	1.00275	newly	34.	200.01	{171., 224.}	488.265	0.766742	Reff	0.629736	0.613751	{0.544651, 0.690735}	6.35978	ar	5934.	9220.71	{8473., 9593.}	55.3878
9	R0	{1.57227, 6.20989}	{1.87551, 12.8355}	{{1.81973, 11.8363}, {1.94812, 14.5138}}	{19.2868, 106.695}	inf	66.	508.701	{446.573, 576.24}	670.759	0.883919	newly	57.	248.28	{224., 272.}	335.579	0.637817	Reff	0.625135	0.623054	{0.594086, 0.644824}	2.81543	ar	6136.	9213.64	{9020., 9556.}	50.1571
10	R0	{1.54936, 6.50938}	{1.81091, 11.1433}	{{1.75086, 10.48}, {1.85189, 11.4548}}	{16.8812, 71.1879}	inf	32.	367.564	{330.93, 388.234}	1048.64	1.05803	newly	31.	206.81	{185., 225.}	567.129	0.8228	Reff	0.582559	0.666143	{0.646808, 0.688298}	14.3726	ar	6275.	9277.48	{8994., 9457.}	47.8483
11	R0	{1.42179, 3.18601}	{2.14847, 16.4501}	{{2.12754, 16.1979}, {2.16678, 16.6971}}	{51.1105, 416.324}	inf	5.	621.86	{603.944, 639.065}	12 337.2	2.09462	newly	7.	235.13	{220., 250.}	3259.	1.52564	Reff	0.665398	0.487529	{0.47784, 0.494219}	26.7311	ar	5322.	10 235.2	{10 160., 10 319.}	92.3179
12	R0	{1.48035, 6.46183}	{1.83821, 13.3849}	{{1.61336, 10.9457}, {1.92709, 14.4397}}	{24.1738, 107.139}	inf	58.	540.114	{404.798, 599.96}	831.232	0.962473	newly	54.	257.8	{229., 283.}	377.407	0.677526	Reff	0.636995	0.692376	{0.657952, 0.755422}	9.96575	ar	5793.	9371.59	{8623., 9746.}	61.7744
13	R0	{1.75755, 8.53854}	{2.23292, 19.8764}	{{2.19997, 20.}, {2.28583, 20.}}	{27.1549, 132.785}	inf	105.	704.22	{664.642, 775.141}	570.686	0.825221	newly	83.	225.22	{202., 253.}	171.349	0.43184	Reff	0.574719	0.450547	{0.433449, 0.468955}	21.8201	ar	6832.	9995.51	{9880., 10 124.}	46.3043
14	R0	{1.47355, 4.89451}	{2.26426, 17.2764}	{{1.73019, 11.8455}, {2.58407, 20.}}	{53.6603, 252.975}	inf	18.	559.769	{353.487, 677.454}	3009.83	1.47787	newly	20.	204.58	{181., 226.}	922.9	1.00832	Reff	0.635542	0.574026	{0.477899, 0.729154}	18.2161	ar	5710.	10 259.5	{8948., 11 043.}	79.6751
15	R0	{1.62071, 5.98665}	{2.00706, 10.6975}	{{1.96178, 10.2709}, {2.04841, 11.1753}}	{23.8383, 78.6888}	inf	12.	314.187	{293.936, 336.519}	2518.23	1.41732	newly	11.	179.49	{165., 199.}	1531.73	1.21142	Reff	0.547962	0.604334	{0.587193, 0.622188}	10.2876	ar	6635.	9858.9	{9742., 10 001.}	48.5893
16	R0	{1.67268, 7.7256}	{1.92612, 12.7457}	{{1.85344, 11.7496}, {1.96872, 13.1991}}	{15.1517, 64.9798}	inf	38.	281.448	{221.109, 322.175}	640.652	0.862015	newly	29.	134.71	{115., 151.}	364.517	0.663797	Reff	0.519367	0.557238	{0.534228, 0.588089}	8.45703	ar	6947.	9399.79	{9197., 9544.}	35.3072
17	R0	{1.67805, 11.1417}	{1.86371, 16.4888}	{{1.73144, 14.4577}, {2.19156, 20.}}	{11.2184, 47.9918}	inf	310.	660.92	{497.7, 861.45}	113.728	0.320539	newly	183.	253.93	{213., 289.}	39.612	0.142793	Reff	0.643364	0.607389	{0.503717, 0.656619}	6.8522	ar	6625.	8798.21	{8303., 9659.}	32.8032
18	R0	{1.66743, 9.99046}	{1.97711, 18.1735}	{{1.6568, 14.5911}, {2.23511, 20.}}	{19.0976, 81.9089}	inf	216.	647.156	{431.575, 795.277}	199.609	0.465033	newly	133.	224.29	{187., 258.}	68.6391	0.223389	Reff	0.574763	0.523252	{0.448449, 0.607381}	12.1805	ar	6899.	9139.97	{8171., 9743.}	32.4825
19	R0	{1.70787, 9.85478}	{1.82409, 19.9525}	{{1.78869, 19.7329}, {1.8982, 20.}}	{6.88551, 102.465}	inf	121.	619.262	{571.44, 660.557}	411.787	0.708303	newly	78.	197.99	{174., 222.}	153.833	0.402792	Reff	0.56428	0.544128	{0.525421, 0.559806}	4.27293	ar	6867.	9235.72	{9073., 9406.}	34.4942
20	R0	{1.53809, 10.5848}	{1.62086, 19.8855}	{{1.55906, 19.4046}, {1.6679, 20.}}	{5.49084, 87.8685}	inf	283.	646.765	{597.453, 687.669}	128.539	0.358276	newly	160.	213.92	{194., 234.}	33.7	0.124984	Reff	0.669684	0.686097	{0.653434, 0.731148}	3.93638	ar	6108.	7950.28	{7672., 8213.}	30.1618
21	R0	{1.59925, 6.74479}	{1.97953, 14.0496}	{{1.82243, 12.651}, {2.35428, 17.9072}}	{23.7784, 108.302}	inf	59.	528.633	{461.275, 641.82}	795.989	0.9468	newly	39.	237.36	{217., 258.}	508.615	0.783153	Reff	0.593482	0.631567	{0.491912, 0.705012}	12.484	ar	6411.	9544.83	{9067., 10 478.}	48.8821
22	R0	{1.38779, 4.40977}	{1.60688, 12.6392}	{{1.50778, 11.3017}, {1.67521, 13.6841}}	{15.7867, 186.619}	inf	49.	383.813	{311.42, 438.17}	683.293	0.888611	newly	54.	193.72	{170., 216.}	258.741	0.552651	Reff	0.682793	0.695602	{0.671788, 0.719681}	2.77141	ar	5154.	7891.91	{7511., 8171.}	53.122
23	R0	{1.53657, 7.50352}	{1.71165, 14.2639}	{{1.56227, 12.2991}, {1.82558, 17.7435}}	{11.3943, 90.0965}	inf	141.	437.232	{314.816, 660.297}	210.094	0.471021	newly	110.	192.55	{151., 248.}	75.0455	0.235177	Reff	0.661647	0.668297	{0.619117, 0.711533}	4.62293	ar	5928.	8434.38	{7902., 9013.}	42.2804
24	R0	{1.71131, 6.24526}	{2.18847, 18.4323}	{{2.16292, 18.1507}, {2.19621, 18.563}}	{27.883, 195.14}	inf	47.	697.187	{675.099, 731.209}	1383.38	1.17092	newly	36.	235.37	{215., 255.}	553.806	0.814472	Reff	0.538036	0.412866	{0.406243, 0.419818}	23.2643	ar	6924.	9987.34	{9859., 10 070.}	44.2423
25	R0	{1.4412, 3.07729}	{2.90637, 20.}	{{2.68515, 20.}, {2.94641, 20.}}	{101.663, 549.922}	inf	2.	788.871	{775.248, 841.281}	39 343.6	2.59582	newly	2.	241.07	{219., 258.}	11 953.5	2.08028	Reff	0.603863	0.276175	{0.264658, 0.340539}	54.2653	ar	5810.	10 807.9	{10 567., 10 903.}	86.0217
26	R0	{1.4159, 4.93858}	{1.90663, 13.9789}	{{1.75908, 12.1645}, {2.12023, 16.2218}}	{34.6586, 183.056}	inf	48.	576.433	{469.444, 702.741}	1100.9	1.07211	newly	55.	263.27	{240., 289.}	378.673	0.678649	Reff	0.683172	0.669401	{0.613878, 0.716084}	5.85647	ar	5255.	9843.92	{9309., 10 638.}	87.3248
27	R0	{1.57465, 7.05414}	{2.00385, 13.4148}	{{1.92811, 12.5746}, {2.05711, 14.9605}}	{27.2568, 90.1693}	inf	116.	603.031	{525.891, 698.786}	419.854	0.713529	newly	88.	283.59	{255., 311.}	222.261	0.506788	Reff	0.599784	0.628846	{0.601868, 0.650633}	4.93625	ar	6359.	9744.96	{9505., 9978.}	53.2467
28	R0	{1.41422, 4.62402}	{2.06482, 15.6219}	{{1.94004, 14.5406}, {2.38356, 18.308}}	{46.0041, 237.842}	inf	11.	786.955	{733.229, 869.835}	7054.13	1.85267	newly	15.	324.62	{297., 347.}	2064.13	1.33432	Reff	0.643046	0.663631	{0.545055, 0.709923}	9.67806	ar	5485.	10 788.4	{10 471., 11 391.}	96.6884
29	R0	{1.41901, 3.63017}	{2.39753, 15.124}	{{2.04671, 12.2383}, {3., 20.}}	{68.9576, 316.619}	inf	5.	532.637	{418.414, 729.783}	10 552.7	2.01356	newly	11.	215.93	{198., 235.}	1863.	1.29205	Reff	0.571577	0.496116	{0.318899, 0.596903}	15.7541	ar	5979.	10 529.3	{9932., 11 488.}	76.1039
30	R0	{1.60719, 4.72984}	{2.97659, 19.0451}	{{2.79522, 17.3978}, {3., 19.4094}}	{85.2049, 302.659}	inf	1.	619.624	{577.659, 634.484}	61 862.4	2.79162	newly	3.	197.13	{181., 213.}	6471.	1.81658	Reff	0.566374	0.257076	{0.247024, 0.263195}	54.6102	ar	6479.	11 057.7	{10 871., 11 131.}	70.6699
31	R0	{1.38175, 6.26468}	{1.75544, 12.1376}	{{1.65166, 10.5377}, {1.9213, 14.6634}}	{27.0445, 93.7464}	inf	50.	393.094	{321.858, 458.944}	686.189	0.889097	newly	41.	204.32	{185., 237.}	398.341	0.69502	Reff	0.648593	0.681858	{0.625554, 0.722285}	6.9107	ar	5397.	9225.16	{8790., 9917.}	70.9313
32	R0	{1.4947, 8.8847}	{1.62107, 18.6506}	{{1.49248, 14.3776}, {1.7033, 20.}}	{8.65391, 109.919}	inf	106.	492.036	{282.85, 605.861}	364.185	0.65006	newly	69.	167.2	{120., 201.}	142.319	0.376431	Reff	0.721193	0.653493	{0.603416, 0.70082}	9.52404	ar	5407.	7797.97	{7072., 8209.}	44.2199
33	R0	{1.55891, 8.28978}	{1.74039, 19.8729}	{{1.64841, 19.4279}, {1.8693, 20.}}	{11.6414, 139.728}	inf	118.	628.678	{562.468, 696.473}	432.778	0.724959	newly	80.	204.14	{176., 230.}	155.175	0.404841	Reff	0.628553	0.61251	{0.536117, 0.671058}	6.3881	ar	6239.	8574.72	{8157., 9005.}	37.4374
34	R0	{1.60764, 10.4729}	{1.77085, 19.5437}	{{1.65552, 19.4049}, {1.93771, 20.}}	{10.7527, 86.6123}	inf	168.	555.499	{472.893, 642.563}	230.654	0.513018	newly	102.	181.08	{148., 217.}	77.5294	0.245159	Reff	0.600936	0.581297	{0.519269, 0.635412}	6.25857	ar	6495.	8735.18	{8278., 9179.}	34.4908
35	R0	{1.48527, 5.74485}	{1.8766, 13.5786}	{{1.77956, 12.6441}, {1.97912, 14.5714}}	{26.3473, 136.361}	inf	59.	580.275	{513.416, 647.157}	883.517	0.990863	newly	58.	276.6	{249., 302.}	376.897	0.677293	Reff	0.640597	0.736288	{0.708483, 0.767102}	14.9378	ar	5811.	9753.69	{9374., 10 121.}	67.8487
36	R0	{1.61457, 9.93364}	{1.83331, 18.3738}	{{1.58838, 14.6418}, {2.00286, 20.}}	{14.2773, 84.965}	inf	204.	653.551	{415.602, 814.747}	220.368	0.49047	newly	131.	223.81	{170., 265.}	70.8473	0.226397	Reff	0.638401	0.573032	{0.496545, 0.647618}	11.1106	ar	6338.	8732.63	{7723., 9394.}	37.7821
37	R0	{1.47831, 7.08139}	{1.66898, 12.6808}	{{1.51567, 10.7954}, {1.79553, 13.6261}}	{12.8976, 79.0716}	inf	93.	389.799	{307.083, 454.84}	319.138	0.616713	newly	79.	196.23	{172., 224.}	148.392	0.393006	Reff	0.683866	0.700485	{0.645726, 0.759263}	5.19161	ar	5530.	8272.57	{7516., 8754.}	49.5944
38	R0	{1.73445, 9.8858}	{2.1177, 17.7452}	{{1.78596, 14.3854}, {2.37942, 20.}}	{22.1555, 79.5018}	inf	168.	681.571	{442.559, 891.207}	305.697	0.592419	newly	85.	240.95	{189., 299.}	183.471	0.446461	Reff	0.583642	0.542558	{0.48019, 0.632038}	10.419	ar	6842.	9652.85	{8818., 10 285.}	41.0823
39	R0	{1.66621, 11.4824}	{1.80659, 18.6423}	{{1.58167, 15.0502}, {1.98883, 20.}}	{10.367, 62.3554}	inf	221.	626.066	{398.475, 792.051}	183.288	0.438529	newly	130.	213.76	{165., 258.}	64.4308	0.210004	Reff	0.657653	0.624306	{0.557075, 0.696713}	7.63083	ar	6473.	8846.07	{7867., 9472.}	36.6611
40	R0	{1.60694, 9.95741}	{1.88262, 19.5515}	{{1.69212, 19.6821}, {1.99818, 20.}}	{17.3115, 96.351}	inf	179.	693.46	{599.569, 789.56}	287.408	0.580916	newly	116.	227.29	{182., 270.}	95.9397	0.287469	Reff	0.592157	0.546476	{0.484917, 0.624747}	10.1794	ar	6625.	9186.85	{8603., 9589.}	38.6694
41	R0	{1.55128, 4.71911}	{2.98599, 19.5496}	{{3., 19.247}, {3., 19.8962}}	{92.4854, 314.265}	inf	16.	713.15	{684.713, 741.053}	4357.19	1.64884	newly	16.	224.16	{206., 244.}	1301.	1.14538	Reff	0.605309	0.303354	{0.287994, 0.310183}	49.8844	ar	6141.	11 422.7	{11 314., 11 511.}	86.0077
42	R0	{1.60063, 6.81118}	{1.92909, 20.}	{{1.91683, 20.}, {1.94289, 20.}}	{20.521, 193.635}	inf	61.	677.814	{661.697, 696.598}	1011.17	1.04562	newly	44.	216.98	{198., 232.}	393.136	0.692037	Reff	0.565983	0.534265	{0.525631, 0.560625}	5.78669	ar	6528.	9615.17	{9501., 9698.}	47.2912
43	R0	{1.56986, 5.44652}	{2.60903, 17.9631}	{{2.04788, 13.0619}, {2.72759, 19.0262}}	{66.1954, 229.809}	inf	36.	688.229	{508.058, 742.784}	1811.75	1.2784	newly	40.	238.92	{218., 262.}	497.3	0.775169	Reff	0.586343	0.459589	{0.423976, 0.584641}	22.1725	ar	6312.	11 235.3	{10 144., 11 532.}	77.999
44	R0	{1.47523, 8.07785}	{1.68676, 15.845}	{{1.57482, 14.403}, {1.80752, 18.2647}}	{14.3389, 96.1541}	inf	154.	512.999	{409.263, 672.328}	233.116	0.514247	newly	118.	208.68	{174., 245.}	76.8475	0.243428	Reff	0.674918	0.662334	{0.607106, 0.708532}	4.58193	ar	5692.	8417.24	{7978., 8964.}	47.8784
45	R0	{1.6417, 7.69429}	{2.17593, 16.9799}	{{1.8051, 12.6248}, {2.51996, 20.}}	{32.5415, 120.682}	inf	70.	574.315	{355.388, 746.794}	720.45	0.894598	newly	45.	210.02	{171., 242.}	366.711	0.665466	Reff	0.547999	0.515819	{0.406173, 0.643232}	18.8019	ar	6735.	9975.36	{9005., 10 783.}	48.1122
46	R0	{1.38865, 5.83473}	{1.63469, 13.6134}	{{1.57372, 12.3146}, {1.68987, 15.7069}}	{17.7178, 133.316}	inf	81.	406.686	{329.995, 588.693}	402.081	0.68867	newly	88.	189.36	{161., 243.}	115.182	0.328319	Reff	0.6778	0.701777	{0.669542, 0.734491}	4.32813	ar	5233.	8035.69	{7775., 8316.}	53.558
47	R0	{1.45289, 6.87903}	{1.61187, 13.0328}	{{1.52633, 10.7829}, {1.69081, 15.2573}}	{10.9422, 89.4575}	inf	113.	420.561	{286.38, 509.569}	272.178	0.562541	newly	105.	205.28	{166., 230.}	95.5048	0.287933	Reff	0.690704	0.699165	{0.662047, 0.729514}	3.38941	ar	5379.	7948.03	{7429., 8291.}	47.7604
48	R0	{1.69416, 9.4831}	{2.06547, 20.}	{{1.93456, 20.}, {2.17672, 20.}}	{21.917, 110.901}	inf	191.	750.157	{691.676, 815.613}	292.752	0.592694	newly	108.	238.56	{205., 274.}	120.889	0.341601	Reff	0.561783	0.496054	{0.464006, 0.539206}	11.7001	ar	6925.	9690.22	{9438., 9886.}	39.931
49	R0	{1.47843, 7.16286}	{1.67768, 13.2297}	{{1.59384, 11.8094}, {1.72522, 15.3462}}	{13.4768, 84.699}	inf	84.	381.907	{317.594, 487.972}	354.651	0.64858	newly	57.	181.25	{158., 208.}	217.982	0.498633	Reff	0.694419	0.672017	{0.640065, 0.702559}	4.00552	ar	5449.	8126.72	{7867., 8400.}	49.1415
50	R0	{1.68843, 5.72427}	{2.05282, 9.9836}	{{1.94887, 8.94924}, {2.21774, 11.4521}}	{21.5818, 74.4082}	inf	27.	191.494	{154.693, 240.212}	609.235	0.845083	newly	30.	114.51	{98., 134.}	281.7	0.578054	Reff	0.541311	0.55413	{0.501497, 0.587987}	5.75341	ar	6800.	9605.86	{9274., 10 058.}	41.2626
